# Ethical Challenges Among Hellenic National Center of Emergency Care (EKAB) Prehospital Emergency Personnel During the Financial Crisis and COVID-19

**DOI:** 10.7759/cureus.114642

**Published:** 2026-08-17

**Authors:** Aristomenis I Syngelakis, Chysoula Tsakalou, Maria Myrto Solomou, Chrystala Charalambous, Aikaterini Toska, Aspasia Goula

**Affiliations:** 1 School of Dentistry, Department of Oral Medicine, European University Cyprus, Nicosia, CYP; 2 Department of Business Administration, Division of Social Policy, University of West Attica, Athens, GRC; 3 General Department of Lamia, University of Thessaly, Greece, Lamia, GRC

**Keywords:** covid-19, emergency medical services, financial crisis, medical ethics, prehospital care

## Abstract

Objective

Τo assess the knowledge, attitudes, and practices of the Hellenic National Center of Emergency Care (EKAB) prehospital emergency personnel regarding ethical and legal issues in their professional practice and to identify the underlying dimensions of ethical perception.

Methods

A validated 32-item structured anonymous online questionnaire was administered to the EKAB prehospital emergency personnel directly involved in patient care. The first part included items on sociodemographic and professional characteristics, while the second part assessed perceptions regarding ethics and deontology in prehospital care. Data collection took place during the later phase of the Greek financial crisis and amidst the COVID-19 pandemic.

Results

A total of 273 participants (50.2% men; mean age range 41-50 years) completed the survey. Exploratory factor analysis (EFA) yielded four factors: (i) autonomy and consent, (ii) resource allocation and triage, (iii) confidentiality vs public health, and (iv) duty and reciprocity. This explained 60.9% of total variance (Kaiser-Meyer-Olkin (KMO) test=0.75; Bartlett’s χ²=1656.44, degrees of freedom (df)=300, p<0.001; α=0.70-0.85). Higher autonomy and consent scores were observed among university-educated staff (p=0.030), and higher duty and reciprocity scores among those >50 years (p=0.022). Significant positive correlations were found between Autonomy and Consent and Confidentiality vs Public Health (ρ=0.522, p<0.01) and between Duty and Reciprocity and Resource Allocation and Triage (ρ=0.465, p<0.01).

Conclusions

EKAB prehospital emergency personnel demonstrate strong ethical commitments across four key domains despite working under dual crisis conditions (economic crisis and the COVID-19 pandemic). These findings provide a framework for targeted ethics education, policy development, and institutional support to strengthen ethical decision-making in prehospital care.

## Introduction

The delivery of prehospital emergency care is inherently shaped by ethical decision-making under conditions of uncertainty, urgency, and limited resources. Paramedics and other prehospital providers must navigate complex situations in which clinical imperatives intersect with ethical principles such as autonomy, beneficence, non-maleficence, and justice. In Greece, this ethical terrain has been further complicated by two major societal stressors in recent years: the prolonged financial crisis that began in 2009 and the coronavirus disease 2019 (COVID-19) pandemic that emerged in early 2020. Both crises have intensified the demands placed on the Hellenic National Center of Emergency Care (EKAB), altering the resources available, the clinical priorities in the field, and the moral pressures on personnel [1,2].

The Greek financial crisis led to sustained austerity measures, reduced public spending, and material shortages in healthcare [1]. For prehospital services, these constraints translated into older ambulance fleets, staff shortages, and limited access to updated medical equipment and protective gear [3]. Such systemic pressures not only compromised operational capacity but also created ethical dilemmas related to triage, equitable allocation of scarce resources, and maintaining quality of care when the means to do so were curtailed. These systemic pressures heightened the relevance of distributive justice, as prehospital providers were frequently required to make rapid decisions about prioritization and resource allocation within an environment of persistent scarcity [3-6].

The onset of the COVID-19 pandemic superimposed a global public-health emergency onto this already fragile context. For EKAB prehospital emergency personnel, the pandemic introduced additional ethical challenges: balancing duty of care with personal safety, applying infection-control measures in uncontrolled environments, and communicating with patients under physical distancing or personal protective equipment (PPE) constraints. Decision-making in the field was further complicated by evolving guidelines, uncertainty about viral transmission risks, and heightened emotional stress, factors known to influence moral judgement and the potential for moral distress among healthcare providers [7-9].

Prehospital settings are distinct from hospital environments in ways that amplify certain ethical tensions. EKAB prehospital emergency personnel often operate with incomplete clinical information, limited time for deliberation, and without direct access to supervising physicians. This makes informed consent more challenging, as patients may be unable or unwilling to engage in detailed discussions due to distress, altered consciousness, or language barriers. Confidentiality, too, may be compromised in the field when care must be delivered in public or semi-public spaces. The ethical principle of beneficence, acting in the patient’s best interest, must often be balanced against non-maleficence when interventions in the field carry risks that cannot be fully explained or mitigated in the moment, a challenge consistently described in emergency medical services (EMS) specific ethical literature [10,11].

Although the literature on healthcare ethics in crisis situations is substantial, much of it focuses on hospital-based or policy-level decision-making. Research on the ethical experiences and attitudes of prehospital emergency providers, particularly in the Greek context, remains scarce. Available studies tend to address discrete topics, such as prehospital triage or infection-control practices, rather than providing a broader understanding of how frontline EMS personnel perceive and navigate ethical and legal issues in daily practice, especially under crisis conditions [12,13]. There is also limited evidence on whether sociodemographic factors (e.g., age, education, professional experience) are associated with differences in ethical perceptions or practices among paramedics.

The Hellenic National Center of Emergency Care (EKAB) plays a central role in Greece’s emergency response system, providing both primary prehospital care and inter-facility transport. EKAB prehospital emergency personnel are thus frequently the first point of contact for critically ill or injured patients and are required to make ethically charged decisions in real time. Understanding their perspectives on ethical and legal issues is essential for developing targeted training, guidelines, and institutional support mechanisms that enhance ethical practice and reduce moral distress. Furthermore, in light of recurring crises, economic, epidemiologic, or otherwise, there is a pressing need for empirical data that can inform the ethical preparedness of prehospital systems.

Against this backdrop, the present study was designed to explore the knowledge, attitudes, and practices of EKAB prehospital emergency personnel regarding medical ethics and health law during the combined stressors of the Greek financial crisis and the COVID-19 pandemic. Specifically, it aimed to: (1) assess the extent to which EKAB prehospital emergency personnel encounter ethical and legal issues in their work; (2) examine their attitudes towards selected bioethical topics, including informed consent, organ donation, and respecting patient wishes; and (3) evaluate how these issues are addressed in practice. By employing a structured questionnaire and psychometric analysis, the study also sought to identify underlying dimensions of ethical perception among EKAB prehospital emergency personnel. The findings may support the design of educational interventions, policy updates, and operational protocols that strengthen ethical decision-making in prehospital care.

This article was previously posted to the Research Square preprint server on October 17, 2025.

## Materials and methods

We conducted a cross-sectional, descriptive study using an online survey to assess the knowledge, attitudes, and practices of EKAB prehospital emergency personnel regarding ethical and legal issues in their professional practice. The study was implemented within EKAB of Greece, which provides primary prehospital care and inter-facility patient transport nationwide. In Greece, prehospital emergency care is provided nationally by the Hellenic National Center of Emergency Care (EKAB), which operates an integrated EMS system staffed primarily by trained prehospital emergency personnel, including paramedics, ambulance crew members, and nurses. Personnel participating in this study reflected these standard operational roles.

Data collection took place from February 15, 2023 to April 30, 2023, during a period spanning the COVID-19 pandemic and the later phase of the Greek financial crisis, both of which had significant operational and ethical implications for the service [1,3,7,8]. The target population comprised personnel employed at EKAB Athens, specifically within the categories of EKAB prehospital emergency personnel and administrative staff. The latter group predominantly consisted of former EKAB prehospital emergency personnel members who had transitioned to administrative posts through internal transfers to equivalent or higher service categories.

Eligibility was defined as current employment in one of these categories during the study period and provision of informed consent to participate. Participants were recruited through internal EKAB communication channels and invited to complete the survey electronically.

The exclusion criteria included personnel who did not have direct clinical or operational involvement in prehospital care (e.g., fleet maintenance staff, administrative staff without patient-related duties), as well as incomplete or duplicate questionnaire responses. Questionnaires with any missing values were excluded entirely from the analysis; partially completed responses were not analyzed, and no data imputation techniques were applied.

Target population and sampling technique

The target population included all EKAB prehospital emergency personnel employed in the Attica region at the time of data collection. The total workforce in this category is estimated at approximately 1,400 staff members, encompassing paramedics, ambulance crew members, nurses, and personnel who provide prehospital emergency services.

A convenience sampling approach was used, consistent with anonymous online surveys conducted within EKAB. An invitation link was disseminated through official internal communication channels, and participation was entirely voluntary. All personnel who met the inclusion criteria were eligible to respond, and no cap on respondent numbers was imposed.

To validate this sampling approach, a post-hoc sample size estimation was conducted for the finite population of 1,400. Using a standard finite population calculation with a 95% confidence level, an estimated population proportion of 0.5, and a 5% margin of error, the target sample size was calculated as 302. The final recruited sample of 273 participants yields a margin of error of approximately 5.3%, which provides acceptable statistical power for the exploratory factor analysis and group comparisons.

Survey tool

A structured questionnaire divided into two parts was used for the study. The first part included items on the sociodemographic and professional characteristics of the participants (e.g. age, sex, marital status, educational level, years of service, work position). The second part consisted of a validated instrument on ethical and legal challenges in clinical practice, originally developed in Greek by Dr. Katerina Toska to explore healthcare professionals’ perceptions of ethics and deontology [14]. Permission for the use of this instrument in its Greek version was formally obtained from the original author, and for the purposes of the present study it was employed without modification.

The full questionnaire contains 32 items. Questions 1-8 record sociodemographic and professional data, while questions 9-32 assess: (a) the perceived frequency with which ethical or legal problems are encountered in daily practice, (b) knowledge of relevant laws and professional codes, and (c) attitudes towards key bioethical topics such as informed consent, confidentiality, truth-telling, end-of-life decision-making, and organ donation. Items in the second part are rated on a five-point Likert scale (from “strongly disagree” to “strongly agree”).

The online instrument also contained an electronic consent checkbox that participants were required to select before proceeding, ensuring voluntary participation. The survey was hosted on a secure online platform and remained open for responses over a defined collection period. Participation was anonymous; no identifying personal or professional data were collected beyond the predefined sociodemographic descriptors. The implementation of the study followed the established official practice for similar studies conducted within EKAB.

Statistical analysis

All analyses were performed using IBM SPSS Statistics version 26.0 (IBM Corp., Armonk, NY, USA). Continuous variables were assessed for normality using the Kolmogorov-Smirnov test. Data are presented as means with standard deviations for normally distributed variables and medians with interquartile ranges for non-normal distributions. Categorical variables are reported as absolute frequencies and percentages.

Between-group differences in continuous or ordinal outcomes were assessed using the Mann-Whitney U test for two-group comparisons and the Kruskal-Wallis H test for comparisons involving more than two groups. Associations between ordinal or continuous variables were evaluated using Spearman’s rank correlation coefficient (ρ).

An EFA with Varimax rotation was performed to identify latent constructs underlying the ethics and legal perception items. Sampling adequacy for factor analysis was confirmed with the Kaiser-Meyer-Olkin (KMO) measure (0.75) and Bartlett’s test of sphericity (p<0.001). Items with factor loadings ≥0.40 were retained in the final factor structure. Internal consistency of each derived factor was assessed using Cronbach’s alpha (α). A two-tailed p-value < 0.05 was considered statistically significant.

Ethics factor scores

Ethics factor scores for each participant were calculated by computing the median value of their responses to the specific items that loaded onto each respective factor. This operational definition was utilized to best accommodate the ordinal nature of the five-point Likert scale data. Because the original items in the second part are rated on a five-point Likert scale (from "strongly disagree" to "strongly agree"), the composite factor scores also range from 1 to 5.

Ethical considerations

The study was conducted in accordance with the principles of the Declaration of Helsinki. All participants provided informed consent through the online consent checkbox before accessing the questionnaire. Participation was voluntary, and no incentives were offered. The survey was anonymous, and data were stored on a password-protected server accessible only to the research team. Ethical approval for the study was granted by the Board of Directors of EKAB following a positive recommendation from the EKAB Scientific Council. This approval was formally documented in Decision No. 3/17.1.2023 and announced through EKAB Administration Document No. 10369/14.2.2023.

## Results

Participant characteristics

A total of 273 EKAB prehospital emergency personnel participated in the study. The key sociodemographic and professional characteristics are presented in Table 1.

**Table 1:** Demographic and professional characteristics of EKAB prehospital emergency personnel (N=273)

Characteristic	n (%)
Gender	
Male	137 (50.2)
Female	136 (49.8)
Age group (years)	
<30	24 (8.8)
31-40	70 (25.6)
41-50	123 (45.1)
>50	56 (20.5)
Educational level	
UL (University Level)	84 (30.8)
TE (Tertiary Education)	68 (24.9)
SE (Secondary Education)	120 (44.0)
CE (Compulsory Education)	1 (0.4)
Years in service	
≤5	13 (4.8)
6-15	78 (28.6)
16-25	118 (43.2)
>25	64 (23.4)

Exploratory factor analysis

An EFA with Varimax rotation was conducted on the ethics and legal perception items. Sampling adequacy was confirmed (KMO=0.75; Bartlett’s test χ²=1656.44, degrees of freedom (df)=300, p<0.001). Four factors with eigenvalues >1 were extracted, explaining 60.9% of the total variance. The factors reflected the following domains: (1) Autonomy and Consent; (2) Resource Allocation and Triage; (3) Confidentiality vs Public Health, and (4) Duty and Reciprocity.

Items with loadings ≥0.40 were retained, and Cronbach’s alpha values ranged from 0.70 to 0.85, indicating acceptable to good internal consistency. Detailed factor loadings and reliability coefficients are presented in Table 2.

**Table 2:** Factor structure of the ethics and legal perception scale [14]. Extraction Method: Exploratory Factor Analysis. Rotation Method: Varimax. Items with factor loadings >0.40 were retained and displayed; cross-loadings below this threshold were suppressed. N=273; Kaiser-Meyer-Olkin (KMO) measure of sampling adequacy=0.75; Bartlett’s test of sphericity chi^2^=1656.44, df=300, p<0.001.

Item	Factor 1: Autonomy & Consent	Factor 2: Resource Allocation & Triage	Factor 3: Confidentiality vs Public Health	Factor 4: Duty & Reciprocity
Obtain consent before interventions	0.72			
Respect patient’s refusal of treatment	0.68			
Ensure patient is informed of all options	0.65			
Allocate resources to most urgent cases		0.74		
Follow triage protocols in mass casualty events		0.69		
Consider fairness when prioritizing care		0.66		
Maintain confidentiality in field settings			0.71	
Share patient data for public health reporting			0.67	
Protect sensitive patient information during emergencies			0.64	
Continue duty despite personal risk				0.76
Expect institutional protection in high-risk work				0.70
Fulfil responsibilities regardless of workload pressures				0.68
Eigenvalue	4.12	3.28	2.45	1.91
Variance explained (%)	25.3	18.2	10.1	7.3
Cumulative variance (%)	25.3	43.5	53.6	60.9
Cronbach’s α	0.85	0.82	0.78	0.70

Group comparisons

Mann-Whitney U and Kruskal-Wallis H tests revealed significant differences in factor scores by age group and educational level. Personnel aged over 50 scored significantly higher on Duty and Reciprocity compared to younger groups (p=0.02). University graduates scored higher on Autonomy and Consent compared to those with non-university education (p=0.030). It should be noted that while this dichotomized comparison (university level (UL) vs. non-UL) yielded statistical significance, analyzing education across all four discrete categories (UL, tertiary education (TE), secondary education (SE), compulsory education (CE)) did not. This discrepancy is primarily attributed to a loss of statistical power caused by highly imbalanced sample sizes in the four-category model (e.g., Compulsory Education, n=1). Dichotomizing the variable into two distinct groups allowed for a more robust evaluation of the effect of university-level training. No significant differences were observed between male and female participants on any factor (p>0.05). Table 3 summarizes the key group comparisons for each ethical dimension.

**Table 3:** Group differences in ethics factor scores by demographic characteristics. Statistical significance was evaluated using the Mann-Whitney U test (denoted by the U statistic) for two-group comparisons, and the Kruskal-Wallis H test (denoted by the H statistic) for multiple-group comparisons. UL=university level; TE=tertiary education; SE=secondary education; CE=compulsory education; IQR=interquartile range.

Factor	Demographic Variable	Group	Median	Interquartile Range (IQR)	Statistic Test	p-value
Autonomy & Consent	Education level	UL (University)	4.6	4.20-4.80	U=2290.5	0.03
		Non-UL	4.4	4.00-4.60	-	-
Duty & Reciprocity	Age group	>50 years	4.7	4.40-4.90	H=7.64	0.022
		≤50 years	4.5	4.20-4.70	-	-
Resource Allocation & Triage	Gender	Male	4.3	4.00-4.50	U=4527.0	0.188
		Female	4.4	4.00-4.80	-	-
	Age group	≤30 years	4.3	4.00-4.80	H=1.66	0.645
		31-40 years	4.3	4.00-4.80	-	-
		41-50 years	4.4	4.00-4.80	-	-
		>50 years	4.3	4.00-4.80	-	-
	Education level	UL	4.3	4.00-4.80	H=0.46	0.926
		TE	4.4	4.00-4.80	-	-
		SE	4.3	4.00-4.80	-	-
		CE	4.3	4.00-4.80	-	-
Confidentiality vs Public Health	Gender	Male	4.5	4.20-4.80	U=4598.0	0.861
		Female	4.5	4.20-4.80	-	-

Correlation analysis

Spearman’s rank correlations indicated moderate positive associations between specific ethical dimensions, as detailed in Table 4.

**Table 4:** Spearman’s rank correlations between ethics factor dimensions

Correlated Pairs	Spearman's rho (ρ)	p-value
Autonomy & Consent - Confidentiality vs Public Health	0.522	<0.01
Duty & Reciprocity - Resource Allocation & Triage	0.465	<0.01

## Discussion

This study provides empirical evidence on the ethical and legal perceptions of EKAB prehospital emergency personnel in Greece during the prolonged financial crisis and the COVID-19 pandemic. Four dimensions of ethical perception, Autonomy and Consent, Resource Allocation and Triage, Confidentiality vs Public Health, and Duty and Reciprocity, emerged from factor analysis, together explaining 60.9% of response variance. The internal consistency of these factors indicates that they represent coherent constructs relevant to prehospital ethical practice.

Autonomy and Consent emerged as the strongest dimension, underscoring the centrality of patient rights even in high-pressure prehospital environments. This aligns with international findings showing that EMS providers prioritize informed consent despite operational time constraints [15,16]. In Greece, the legal framework reinforcing informed consent [17] may further strengthen this emphasis. University-educated participants scored higher on this factor, consistent with research linking academic training to increased ethical sensitivity in prehospital care.

Resource Allocation and Triage also featured prominently, reflecting the ethical significance of distributive justice in EMS. Median scores were high across the sample, indicating a broadly shared commitment to fairness during resource scarcity. This is consistent with literature on triage in mass-casualty incidents and pandemics [18,19], as well as international ethical guidance emphasizing fairness and transparency in crisis response [20,21]. Despite the chronic resource constraints associated with the Greek financial crisis, EKAB personnel in this study appeared to uphold these norms, suggesting a resilient ethical orientation.

The third factor, Confidentiality vs Public Health, captures tension between privacy protection and public health obligations, an issue heightened during COVID-19 due to contact tracing and mandatory reporting requirements [22]. The observed correlation with Autonomy and Consent suggests that personnel who value patient autonomy also tend to emphasize privacy protection. Managing these tensions requires clear communication and institutional safeguards to maintain patient trust, a key determinant of compliance during public health emergencies [23-26].

Duty and Reciprocity reflect professional obligation in the face of personal risk and was endorsed more strongly by older participants. This may reflect generational differences in role identity or experience with high-risk scenarios [7,8]. The association between this factor and Resource Allocation suggests that a strong sense of duty may reinforce willingness to make difficult triage decisions. However, the literature cautions that duty without adequate institutional reciprocity, such as protection, support, and recognition, can contribute to moral injury and burnout [27].

Overall, these findings align with international EMS ethics research while highlighting the additional strain imposed by Greece’s long-term economic austerity [4]. The consistently high scores across domains may reflect a strong personal and professional commitment among EKAB personnel, although the cumulative impact of dual crises underscores the importance of organisational support to sustain ethical practice. Ethics training, clear crisis-response guidelines, and psychosocial support mechanisms may help mitigate moral distress and reinforce ethical resilience [28,29].

Health policy implications

The four ethical dimensions identified can inform the development of targeted ethics education and EMS policy. Training aligned with autonomy, fairness in allocation, confidentiality management, and duty-of-care expectations may strengthen preparedness for both routine operations and crises. Clear guidance on privacy during public health emergencies and structured ethics debriefings may further support workforce well-being and ethical consistency [28-30].

Strengths and limitations

The strengths of the study include the use of a validated, psychometrically assessed instrument, a relatively large sample, and the application of factor analysis to identify latent ethical constructs.

The limitations of this study include the cross-sectional design, which precludes causal inferences, and reliance on self-reported measures, making the study particularly susceptible to social desirability bias where respondents may overestimate their ethical commitment. Furthermore, the use of a convenience sampling method and the absence of exact response rate tracking introduce potential selection bias. Because the sample predominantly comprised personnel from the Attica region, the findings may have limited generalizability to EMS systems in other regions of Greece or internationally. The online format may have excluded less digitally literate staff, and recall bias is possible given that data were collected three years after the onset of COVID-19. Finally, the ethical domains identified using exploratory factor analysis provide an exploratory framework that requires validation through confirmatory factor analysis (CFA) in independent samples to ensure structural stability.

Future research

Future studies should examine how these ethical dimensions evolve, particularly in response to changes in crisis intensity or healthcare policy. Qualitative research could complement these findings by exploring how EKAB prehospital emergency personnel interpret and apply ethical principles in specific, high-stakes incidents. Comparative studies between Greek EMS and services in other crisis-affected countries could further illuminate cultural and systemic influences on ethical decision-making.

## Conclusions

Prehospital emergency providers in Greece operate at the intersection of urgent clinical care and complex ethical decision-making. This study, conducted during the overlapping pressures of a prolonged financial crisis and a global pandemic, identifies four key domains of ethical perception: Autonomy and Consent, Resource Allocation and Triage, Confidentiality vs Public Health, and Duty and Reciprocity. These domains capture the core tensions that paramedical personnel face when balancing patient rights, equitable care, public health responsibilities, and personal risk.

The findings demonstrate that, despite severe systemic constraints, EKAB prehospital emergency personnel report a strong perceived commitment to fundamental ethical principles. It is important to note that these findings reflect self-reported attitudes rather than objectively measured clinical behaviours. However, they highlight areas where targeted training, institutional support, and clear policy frameworks could further strengthen ethical practice and reduce moral distress. While the exploratory framework of ethical domains identified here requires further multicenter validation before widespread policy implementation, integrating these preliminary concepts into EMS education and operational planning can assist prehospital systems in building resilience against future crises and ensure that ethical integrity remains central to emergency care delivery.
